# Immediate and delayed effects of thermal stress on fever-associated seizures in children: A time-stratified case-crossover study in Japan

**DOI:** 10.1007/s00484-026-03146-z

**Published:** 2026-03-05

**Authors:** Naomi Matsumoto, Yuka Yamamura, Kensuke Uraguchi, Takafumi Obara, Hiromichi Naito, Takashi Yorifuji

**Affiliations:** 1 Department of Epidemiology, Faculty of Medicine, Dentistry and Pharmaceutical Sciences, Okayama University, 2-5-1 Shikata-Cho, Kita-Ku, Okayama, Japan; 2 Department of Epidemiology, Okayama University Graduate School of Medicine, Dentistry and Pharmaceutical Sciences, Okayama, Japan; 3 Department of Otolaryngology-Head & Neck Surgery, Kagawa Rosai Hospital, Kagawa, Japan; 4 Department of Emergency, Critical Care, and Disaster Medicine, Okayama University Faculty of Medicine, Dentistry, and Pharmaceutical Sciences, Okayama, Japan

**Keywords:** Time-stratified Case-crossover study, Thermal stress, Fever-associated seizures, Universal Thermal Climate Index (UTCI), Climate change, Pediatric emergency

## Abstract

This study aimed to examine the non-linear and delayed effects of thermal stress, measured by the hourly Universal Thermal Climate Index (UTCI), on the risk of pediatric fever-associated seizures (FAS). We conducted a time-stratified case-crossover study in Okayama, Japan (May 2015–March 2023), analyzing 3,201 ambulance-attended FAS cases in children younger than 7 years. Using a distributed lag non-linear model (DLNM) with a 144-h lag, we estimated the association between UTCI and FAS. The analysis revealed a bimodal exposure–response relationship. Moderate Cold Stress (10th percentile, –1.6 °C) was associated with a significant cumulative odds ratio (OR) of 2.22 (95% CI: 1.22–4.06). Risk also increased at the upper range of No Thermal Stress (24.2 °C; cumulative OR 2.74, 95% CI: 1.63–4.63), extending into Moderate Heat Stress (28.7 °C; cumulative OR 2.26, 95% CI: 1.33–3.84). These effects were primarily delayed to 72–96 h for Moderate Cold and reached a peak around 100 h for Moderate Heat. Strong Heat Stress showed immediate but non-significant risk patterns. These findings suggest that infection-mediated pathways likely drive the observed bimodal risk pattern, demonstrate the utility of high-resolution bioclimatic indices, and can inform the development of temperature-specific public health alerts.

## Introduction

Thermal stress is a well-recognized determinant of morbidity and mortality (Gasparrini et al. 2015). Children are particularly vulnerable to both heat and cold due to their physiological immaturity, higher surface area-to-mass ratio, and limited behavioral adaptation (Azan et al. 2025). Recent research has established child-specific thermal benchmarks, suggesting a neutral Universal Thermal Climate Index (UTCI) of 13.9 °C and specific thermal safety thresholds for pediatric populations (Huang et al. 2021). In the Western Pacific, including Japan, epidemiological analyses attribute a substantial burden to non-optimal temperatures—predominantly from moderate cold for mortality—while heat contributes importantly to morbidity (Zhao et al. 2021; Yuan et al. 2024). UNICEF warns that 65% of children in this region face four or more overlapping climate-related hazards, including heatwaves, compared with a global average of 37% (Joshi et al. 2023). Evidence from Aotearoa New Zealand shows J-shaped temperature–admission curves, with risks increasing above 24.1 °C and greater sensitivity among Māori, Pacific, and Asian children than among European children (Lai et al. 2024). A multi-country study found Japan had one of the highest mortality fractions from non-optimal temperatures (≈10%), driven mainly by moderate cold, highlighting the high thermal vulnerability of East Asian populations (Gasparrini et al. 2015).

Fever-associated seizures (FAS) are a common and observable manifestation of such thermal vulnerability in children. Among them, febrile seizures (FSs), the most frequent subtype of FAS, are defined as seizures with fever (≥ 38 °C) in children aged 6 months to 5 years without central nervous system infections or other underlying conditions. FSs affect about 4% of children worldwide (Woo et al. 2018), recur in roughly one-third of cases, (Leung et al. 2018) and cause parental anxiety, frequent emergency visits, and represent a substantial burden on families and healthcare systems (Kim et al. 2017; Jiang et al. 2024). Triggers are complex, including genetic predispositions, viral infections, and possibly meteorological factors such as ambient temperature (Millichap and Millichap 2006). Japan is largely characterized by a humid subtropical climate (Cfa), featuring high humidity and distinct, rapid temperature shifts during the transitional seasons. These humidity-driven heat stresses and seasonal fluctuations may uniquely sensitize the pediatric population to thermal changes. In Japan, only a single-center observational study examined weather and infectious disease epidemics as potential triggers for febrile seizures, and it did not demonstrate a significant effect of meteorological factors after accounting for epidemic trends (Kawakami et al. 2020).

A growing body of epidemiological evidence suggests associations between meteorological conditions and the incidence of pediatric seizures, particularly in East Asian regions (Woo et al. 2018; Jiang et al. 2024; Fan et al. 2024). While some identified increased risks during colder periods, (Kim et al. 2017; Jiang et al. 2024) recent studies, often employing robust time-stratified case-crossover designs combined with distributed lag non-linear models (DLNMs), (Gasparrini et al. 2010) have suggested complex, non-linear temperature-seizure relationships, such as U-shaped or inverted J-shaped patterns (Woo et al. 2018; Jiang et al. 2024; Zhang et al. 2024). These findings also indicate that both cold and heat can affect seizure risk, with effects that may be delayed and vary in intensity over time (Woo et al. 2018; Zhang et al. 2024). The variation in these findings across different regions suggests that regional climatic characteristics and population susceptibility modify temperature effects on fever-associated seizures in children.

These inconsistent findings and the inability to disentangle direct meteorological effects from seasonal infectious disease trends, potentially stem from two critical methodological limitations. First, prior research predominantly used simple temperature indicators (e.g., daily mean air temperature) rather than more physiologically relevant thermal indices, such as the Universal Thermal Climate Index (UTCI). The UTCI integrates multiple meteorological factors to model human thermoregulatory responses more accurately (Jendritzky et al. 2012; Błażejczyk et al. 2013; Pappenberger et al. 2015; Romaszko et al. 2022). Second, most analyses were limited to daily or weekly temporal aggregations, potentially missing critical short-term exposure–response windows, such as those triggered by rapid intraday temperature shifts or short-term extreme peaks (Guo et al. 2011; Woo et al. 2018; Jiang et al. 2024; Zhang et al. 2024). Acute physiological responses to heat stress, such as thermoregulatory dysfunction, inflammatory cascade activation, and increased susceptibility to infection-related fever spikes, (Lakhoo et al. 2025) can occur rapidly, and coarse temporal resolution may thus mask or misestimate the associations. Zhu et al. analyzed hourly temperature effects on acute ischemic stroke with a 24-h lag period by combining the DLNM with a case-crossover design (Zhu et al. 2024). This hourly approach showed acute exposure–response patterns missed by daily analyses, and similar benefits are expected in research on pediatric seizures.

Therefore, this study aimed to address these important knowledge gaps by investigating the association between hourly UTCI exposure and fever-associated seizures in children < 7 years, using a time-stratified case-crossover design with the DLNM. The 144-h (6-day) lag period was established to evaluate comprehensively immediate physiological effects of heat stress (0–24 h) and delayed effects mediated by viral infections (1–6 days including infection incubation periods). By leveraging comprehensive Japanese emergency transport data, our findings aim to improve understanding of cold and heat stress effects in susceptible pediatric populations in East Asia, providing crucial evidence for targeted prevention strategies and informing climate–health adaptation planning in the context of global climate change.

## Methods

### Study design and population

We conducted a time-stratified case-crossover study to investigate the association between thermal stress exposure and fever-associated seizures in children. The study population included children (approximately 300,000 child-years of observation) in Okayama City and the neighboring town of Kibi-Chuo, located in western Japan. The region belongs to the humid subtropical climate (Cfa) zone and is specifically characterized by the Setouchi climate, which features a high number of annual sunshine hours and low precipitation. Okayama City is a coastal urban center, while Kibi-Chuo Town is located in a hilly inland area. These geographical differences influence local meteorological parameters—such as air temperature, humidity, and solar radiation—which are collectively reflected in the UTCI. We used ambulance dispatch records from 1 May 2015 to 31 March 2023. This dataset is considered representative of the entire pediatric population in the area, as Japan’s universal healthcare system provides emergency ambulance services free of charge, minimizing socioeconomic barriers to access. The case-crossover design is well-suited for this research because it inherently controls for time-invariant individual confounders, such as sex, age, and underlying comorbidities, by using each case as its own control (Guo et al. 2011; Cheng et al. 2022; Zhu et al. 2024).

### Cases of fever-associated seizures in children

We identified cases of FAS in children younger than 7 years from an ambulance dispatch dataset. A case was defined by emergency medical service (EMS) records as a child with: (i) the chief complaint of seizures and (ii) a body temperature ≥ 38.0 °C. This broader age category was chosen to capture fever-related convulsive events occurring beyond the typical 6-month to 5-year peak incidence period of traditional febrile seizures. All patients presenting with fever and seizures were included, regardless of their specific provisional diagnosis (e.g., heat-stroke) given the uncertainty inherent in provisional diagnoses. Patients with missing information on these key variables were excluded.

### Exposure assessment

To assess thermal stress, we used the Universal Thermal Climate Index (UTCI). The UTCI is an advanced thermal index based on human heat balance modeling that represents physiological strain more accurately than simple temperature metrics by integrating four key meteorological variables: 2-m air temperature (Ta), 2-m relative humidity, 10-m wind speed, and mean radiant temperature (MRT) (Jendritzky et al. 2012; Błażejczyk et al. 2013). The UTCI categorizes thermal stress as follows: Strong Cold Stress (− 13 to − 27 °C), Moderate Cold Stress (− 13 to 0 °C), Slight Cold Stress (0 to 9 °C), No Thermal Stress (9 to 26 °C), Moderate Heat Stress (26 to 32 °C), Strong Heat Stress (32 to 38 °C), and Very Strong Heat Stress (> 38 °C) (Błażejczyk et al. 2013).

Hourly data for these variables were obtained from the ERA5-Land reanalysis dataset, provided by the Copernicus Climate Data Store, which features an enhanced spatial resolution of approximately 9 km (Hersbach et al. 2020; Muñoz-Sabater et al. 2021). In this study, UTCI was calculated using an estimation of MRT based on radiant fluxes. Specifically, hourly surface solar and thermal radiation fluxes (*W/m*^*2*^) were derived from cumulative ERA5-Land variables by calculating the difference between successive time steps. MRT was estimated by incorporating these fluxes along with air temperature to account for radiant heat load.

Exposure was assigned by linking these hourly meteorological data to each case's location. We identified representative geographic coordinates for each of the 101 elementary school districts within the study area using school location data from the National Land Numerical Information database. These data were processed using QGIS (version 3.36). For closed schools or dispatch records that covered areas larger than a single school district, coordinates were manually determined using Google Maps or by averaging the locations of relevant original schools. Exposure for each case was then assigned based on data from the nearest ERA5-Land grid point to these coordinates. The final UTCI value for each hour and location was calculated using the “pythermalcomfort” Python library (Tartarini and Schiavon 2020).

### Study design specifics: case and control definitions

We implemented a time-stratified design to select control periods, which is a strategy that minimizes bias from time trends in environmental exposures (Zhu et al. 2024). The case period for each patient was defined as the specific hour of the ambulance dispatch for each seizure event.

Control periods for each case were defined as the same hours on all other same days of the week, within the same calendar month and year. For example, if a case occurred on a Monday at 3 PM in July 2020, the control periods would be all other Mondays at 3 PM in July 2020. This matching strategy robustly controls for confounding by the day of the week, time of day, and seasonality.

### Statistical analysis

We conducted a descriptive analysis to summarize the characteristics of cases and the corresponding meteorological conditions at the time of the event. Demographic characteristics of the patients (sex and age) were summarized as counts and percentages. Key meteorological variables (UTCI, air temperature, relative humidity, and wind speed) are presented as medians with interquartile ranges (IQRs). These summaries were calculated for the entire study period and stratified by season (spring: March to May; summer: June to August; autumn: September to November; winter: December to February).

We used a time-stratified case-crossover design with conditional logistic regression to examine the association between the UTCI and fever-associated seizures in children. We applied DLNMs to capture non-linear and delayed effects of temperature exposure. This framework allows simultaneous estimation of exposure–response and lag–response relationships. This model, which was conditional on each case–control stratum, included a cross-basis function for the UTCI with a maximum lag of 144 h (6 days) to include immediate and delayed effects. We adjusted for public holidays as a binary covariate to account for potential changes in healthcare-seeking behavior. Air pollutants (e.g., *PM*_*2.5*_) were not included in the primary model to avoid potential over-adjustment bias.

The exposure–response and lag–response relationships were modeled using natural cubic splines. The optimal degrees of freedom (df) were selected by minimizing the Akaike information criterion over a range of 3 to 5 for both dimensions, which resulted in 4 for the exposure–response and 3 for the lag–response. In the exposure–response spline (df = 4), internal knots were automatically placed at 4.7 °C, 14.7 °C, and 24.2 °C according to the distribution of the UTCI. In the lag-response spline (df = 3), an internal knot was automatically placed at 72 h on the lag scale, as determined by the software’s automatic knot placement procedure. The reference UTCI value was set at 13.9 °C, representing the “neutral UTCI” for children as suggested by Huang B. et al. (Huang et al. 2021). To demonstrate the robustness of this selection, we performed a sensitivity analysis using 20.0 °C as an alternative reference point, representing the 'Preferred UTCI' for children identified in the same study. We calculated odds ratios (ORs) with 95% confidence intervals (CIs) for specific UTCI percentiles (5th, 10th, 25th, 50th, 75th, 90th, 95th, and 99th) relative to the reference value, and examined lag-specific and cumulative effects over the entire lag period.

We conducted sensitivity analyses to assess the robustness of our findings. Alternative knot placements for the exposure–response relationship were tested by fixing knots at the 10th, 50th, and 90th percentiles of the UTCI distribution. Although stratified analysis by season was pre-specified, these models showed statistical instability because of limited within-strata sample sizes. Therefore, we adopted a descriptive approach and visualized the distribution of cases across UTCI values for each season (summer or winter) using box plots overlaid on the overall exposure–response curve.

Data processing and statistical analyses were performed using Python (version 3.11; Python Software Foundation, Wilmington, DE, USA) with pandas and netCDF4 libraries for retrieving ERA5 data and calculating the UTCI. Stata (version 18.5; StataCorp LLC, College Station, TX, USA) was used for data management and descriptive analyses, and R (version 4.5.0; R Foundation for Statistical Computing, Vienna, Austria) with the “dlnm” and “survival” packages was used for DLNM modeling. Statistical significance was set at *α* = 0.05 for two-sided tests.

## Results

### Study population and case selection

**Fig. 1:**
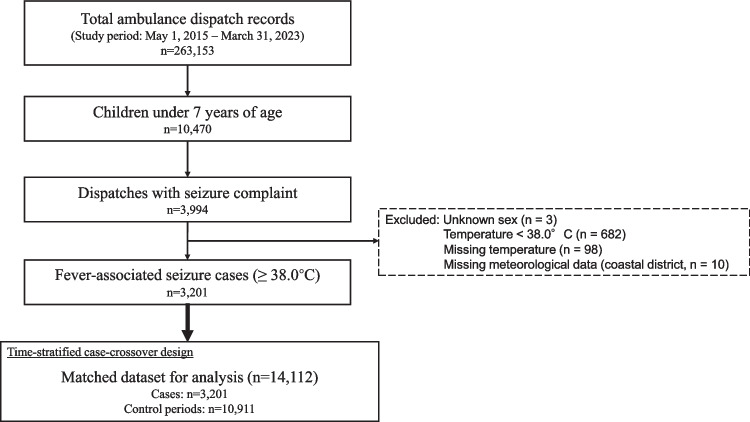
Flowchart of case selection

The case selection process is shown in Fig. 1. From an initial 263,153 ambulance dispatch records, we included a final analytical dataset of 3,201 pediatric cases of fever-associated seizures (FAS). Cases were primarily excluded if they did not meet the age criteria (< 7 years), if seizures were not the chief complaint, or because of missing data (e.g., body temperature). We also excluded cases with a body temperature < 38.0 °C. Additionally, ten cases from a single coastal elementary school district were excluded because of unavailable corresponding meteorological data (0.31% of 3,211 total cases). Of the final cases, two (0.06%) had a provisional diagnosis of heat stroke. In the time-stratified case-crossover analysis, the 3,201 cases were matched with 10,911 control periods (same hour of the day, same day of the week, within the same month and year), resulting in 14,112 observations.

### Characteristics of FAS cases

Among the 3,201 FAS cases with fever, 58.8% (*n* = 1,882) were boys. The majority were 1 year of age (46.9%, *n* = 1,501), followed by 2 years of age (22.4%, *n* = 717). The seasonal distribution showed that 30.5% (*n* = 977) of cases occurred during summer and 27.9% (*n* = 894) during winter. The remaining 41.6% (*n* = 1,330) occurred during the transitional seasons of spring and autumn (Table 1).

**Table 1:** Characteristics of fever-associated seizure cases and meteorological conditions according to the season

	Season				
	Total (*n* = 3,201)	Spring (*n* = 651)	Summer (*n* = 977)	Autumn (*n* = 679)	Winter (*n* = 894)
Characteristics of cases					
Sex					
Boys	1,882 (58.8%)	372 (57.1%)	576 (59.0%)	398 (58.6%)	536 (60.0%)
Girls	1,319 (41.2%)	279 (42.9%)	401 (41.0%)	281 (41.4%)	358 (40.0%)
Ages					
0	232 (7.2%)	47 (7.2%)	77 (7.9%)	49 (7.2%)	59 (6.6%)
1	1,501 (46.9%)	315 (48.4%)	500 (51.2%)	322 (47.4%)	364 (40.7%)
2	717 (22.4%)	138 (21.2%)	206 (21.1%)	165 (24.3%)	208 (23.3%)
3	358 (11.2%)	64 (9.8%)	111 (11.4%)	72 (10.6%)	111 (12.4%)
4	197 (6.2%)	43 (6.6%)	43 (4.4%)	51 (7.5%)	60 (6.7%)
5	123 (3.8%)	28 (4.3%)	25 (2.6%)	14 (2.1%)	56 (6.3%)
6	73 (2.3%)	16 (2.5%)	15 (1.5%)	6 (0.9%)	36 (4.0%)
Meteorological conditions					
UTCI (°C)	14.7 (4.7, 24.2)	12.1 (6.4, 17.4)	26.7 (23.1, 29.5)	16.9 (11.6, 22.3)	1.2 (−3.0, 5.6)
2 m Temperature (°C)	15.3 (7.3, 22.9)	12.7 (8.6, 16.7)	24.4 (21.9, 26.5)	17.2 (13.0, 21.7)	5.0 (2.7, 7.6)
2 m Relative humidity (%)	81.4 (70.4, 89.2)	81.7 (70.2, 89.2)	87.5 (79.8, 93.1)	81.7 (71.1, 87.8)	73.4 (63.4, 82.1)
10 m wind speed (m/s)	2.2 (1.5, 3.3)	2.0 (1.3, 3.0)	1.9 (1.2, 2.8)	2.3 (1.7, 3.3)	2.8 (1.8, 4.1)

Data are presented as n (%) for categorical variables and the median (25th–75th percentiles) for continuous variables

*UTCI* Universal Thermal Climate Index

The UTCI (median, p25, p75) was calculated from air temperature, relative humidity, wind speed, and mean radiant temperature (assumed to be equal to air temperature)

Spring: March to May; Summer: June to August; Autumn: September to November; Winter: December to February

Percentages indicate the proportion of total cases in each season

The meteorological conditions at the time of case occurrence varied substantially by season. The median UTCI was 14.7 °C (IQR: 4.7, 24.2) for the entire study period, with marked seasonal variation: median UTCI was 26.7 °C (IQR: 23.1–29.5) in summer and 1.2 °C (IQR: −3.0–5.6) in winter. Relative humidity was higher in summer (median: 87.5%) than in winter (median: 73.4%), while wind speed was stronger in winter (median: 2.8 m/s) than in summer (median: 1.9 m/s), reflecting the humid subtropical climate of the study area (Table 1).

### Association between the UTCI and fever-associated seizures in children

**Fig. 2:**
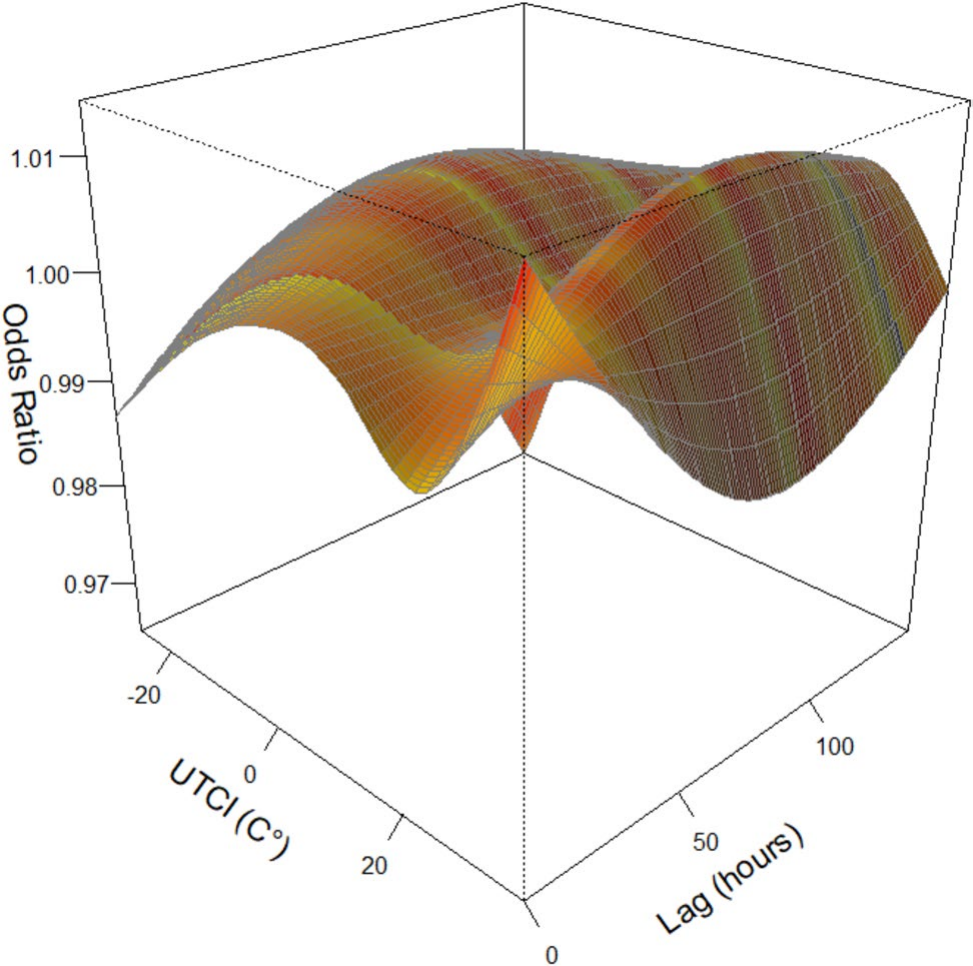
Three-dimensional visualization of the UTCI–lag–response relationship. The three-dimensional surface plot shows the association between hourly UTCI, lag time *(0–144 h)*, and odds ratios (ORs) for FAS. The x-axis represents hourly UTCI, the y-axis represents lag time, and the z-axis represents ORs. Reference UTCI: 13.9 °C

The three-dimensional visualization of the UTCI–lag–response relationship showed complex temporal patterns across the entire exposure range (Fig. 2).

**Fig. 3:**
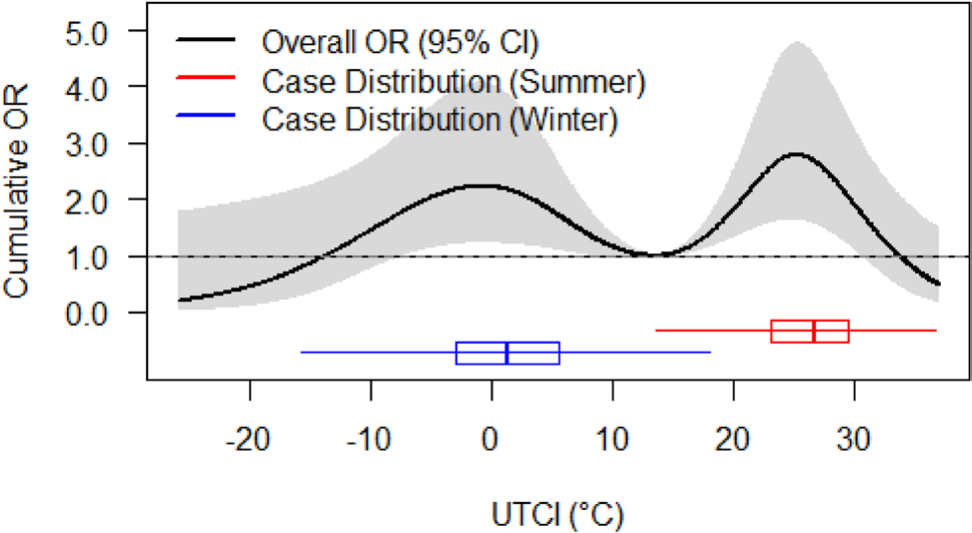
Overall cumulative exposure–response relationship with seasonal case distribution. The black line represents the cumulative odds ratios (ORs) over 144 h with 95% confidence intervals (gray area). Reference UTCI: 13.9 °C. Overlaid box plots represent case distributions for winter (blue, median UTCI: 1.2 °C) and summer (red, median UTCI: 26.7 °C)

The cumulative exposure–response relationship over 144 h showed a bimodal association between temperature and the risk of FAS (Fig. 3 and Table 2). There was a cold-related peak at the 10th percentile (− 1.6 °C; OR: 2.22, 95% CI: 1.22–4.06) and a warm-related peak at the 75th percentile (24.2 °C; OR: 2.74, 95% CI: 1.63–4.63), with a reference temperature of 13.9 °C. Additional significant associations were observed at the 5th percentile (− 5.4 °C; OR: 2.00, 95% CI: 1.11–3.58) and the 90th percentile (28.7 °C; OR: 2.26, 95% CI: 1.33–3.84). Strong heat exposure at the 99th percentile (33.1 °C) showed no significant association with the risk of seizures (OR: 1.12, 95% CI: 0.56–2.25).

**Table 2:** Cumulative odds ratios of fever-associated seizures at specific UTCI percentiles over the 144-h lag period

UTCI Percentile	UTCI (°C)	Odds Ratio	95% Confidence Interval	P-value
5th	−5.4	2.00	(1.11, 3.58)	0.02
10th	−1.6	2.22	(1.22, 4.06)	0.01
25th	4.7	1.83	(1.12, 2.98)	0.02
50th	14.7	1.03	(0.99, 1.06)	0.13
75th	24.2	2.74	(1.63, 4.63)	0.00
90th	28.7	2.26	(1.33, 3.84)	0.00
95th	30.6	1.75	(1.00, 3.05)	0.05
99th	33.1	1.12	(0.56, 2.25)	0.75

Odds ratios are presented as cumulative effects over the entire 144-h lag period relative to the reference UTCI value of 13.9 °C

*UTCI* Universal Thermal Climate Index

### Lag-specific effects

**Fig. 4:**
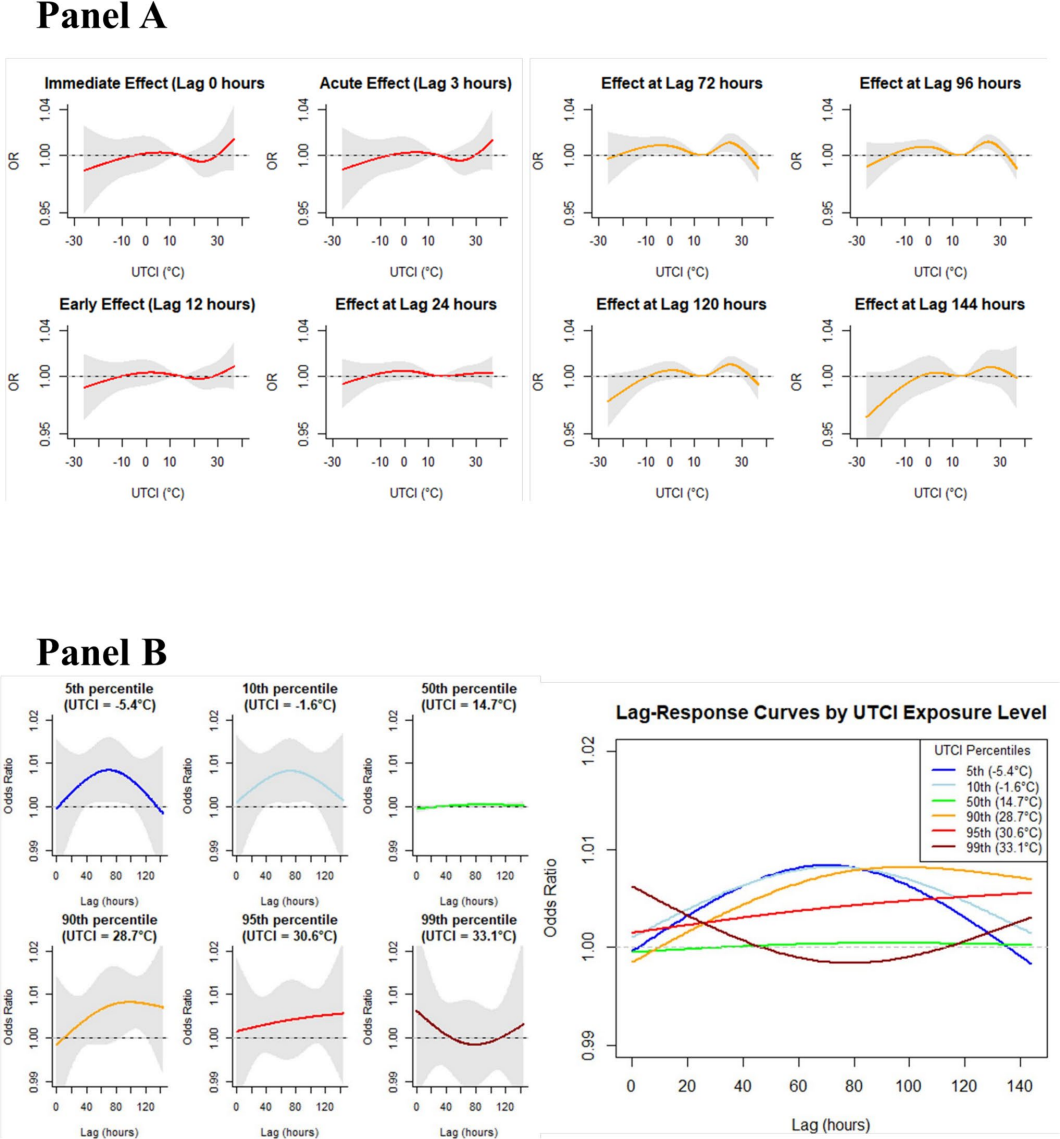
Lag-specific and UTCI-specific lag–response curves. **A**: Exposure–response curves at specific lags showing immediate to delayed effects. (0–144 h). **B**: Lag-response curves for specific UTCI percentiles. Colors correspond to: 5th (blue, −5.4 °C), 10th (light blue, −1.6 °C), 50th (green, 14.7 °C), 90th (yellow, 28.7 °C), 95th (red, 30.6 °C), and 99th (dark red, 33.1 °C). Left: UTCI-specific lag-response curves over 144 h (5-99th percentiles). Right: Overlaid lag–response curves for UTCI percentiles by exposure levels

Lag-specific analysis showed distinct temporal patterns for cold, warm and hot conditions (Fig. 4A). For Moderate Cold and Moderate Heat exposure, no significant lag-specific effect was observed within the first 24 h, but risk progressively increased and reached a maximum lag-specific effect, around 72–96 h for Moderate Cold and 72–120 h for Moderate Heat. In contrast, no such delayed effects were observed for Strong Heat or near the neutral UTCI (13.9 °C).

### UTCI-specific lag–response curves

The analysis of lag–response curves at different UTCI percentiles showed temperature-dependent patterns (Fig. 4B). Moderate Cold Stress (5th and 10th percentile, − 5.4 °C and −1.6 °C) showed significant associations at 72–96 h, and Moderate Heat Stress (28.7 °C, 90th percentile) similarly demonstrated a significant delayed effect peaking around 100 h (Fig. 4B). Strong Heat Stress (33.1 °C, 99th percentile) showed a transient immediate trend within 0–24 h, though it did not reach statistical significance.

### Seasonal distribution of cases

The seasonal distribution of cases overlaid on the overall cumulative exposure–response curve showed distinct patterns (Fig. 3). Winter cases were predominantly distributed in the Moderate Cold Stress range (median UTCI: 1.2 °C), coinciding with the first risk peak. Summer cases were concentrated in the upper range of No Thermal Stress and within Moderate Heat Stress (median UTCI: 26.7 °C), aligning with the second, higher risk peak. Notably, the risk attenuated at both thermal extremes, specifically when the UTCI dropped below 0 °C or exceeded 30 °C. These patterns suggest that FAS risk is highest during the most frequent thermal exposures of summer and winter, rather than during extreme heat or cold events.

### Sensitivity analysis

The sensitivity analysis with fixed knot placement at the 10th, 50th, and 90th percentiles of the UTCI distribution showed substantially wider confidence intervals than those in the main analysis, particularly at extreme heat (data not shown). This instability suggests that the data-driven knot selection (df = 4) was more appropriate for our dataset. While the qualitative patterns remained consistent with the main analysis, the magnitude of effect estimates varied with different knot placements. Additionally, a sensitivity analysis using the "Preferred UTCI" (20.0 °C) (Huang et al. 2021) as an alternative reference point preserved the bimodal association, with the primary risk peaks remaining robust (data not shown). These findings confirm that our results are independent of specific model parameters or reference point selection.

## Discussion

Our time-stratified case-crossover study revealed a distinct bimodal association between hourly thermal stress, measured by the UTCI, and FAS. The cumulative risk over 144 h was elevated in both the colder and warmer ranges of the thermal spectrum, with the highest risk observed at the 75th percentile of the UTCI (24.2 °C, OR 2.74). Between these peaks, the risk was significantly lower near the neutral UTCI (13.9 °C), confirming the bimodal nature of the relationship. Both cold and heat peaks were primarily characterized by a delayed effect (72–120 h, with Moderate Cold peaking at 72–96 h and Moderate Heat at around 100 h). This specific lag pattern is consistent with the incubation periods of common seasonal viruses, strongly suggesting that the observed bimodal risks are driven by infection-mediated pathways rather than direct thermoregulatory failure. In contrast, the risk attenuated during Strong Heat Stress and more severe cold, possibly reflecting both a limited number of extreme events (reducing statistical power) and exposure modification such as avoidance behaviors.

Our results align with previous East Asian studies (Kim et al. 2017; Woo et al. 2018; Jiang et al. 2024; Zhang et al. 2024). Our finding of a bimodal association between UTCI exposure and fever-associated seizures with two distinct peaks is partially consistent with the U-shaped association reported in the Seoul study, (Woo et al. 2018) and the delayed effect of cold exposure (72–96 h) aligns with the findings from the Shanghai study, (Zhang et al. 2024) which used a daily resolution. Our analysis using the hourly UTCI was unique in suggesting a transient immediate trend during Strong Heat Stress, although it did not reach statistical significance, which was previously undetected in daily analyses. This distinction may reflect the enhanced sensitivity of UTCI or the improved temporal resolution of our hourly analysis (Jendritzky et al. 2012; Błażejczyk et al. 2013; Zhu et al. 2024).

The distinct temporal patterns observed for cold exposure in our study suggest different underlying mechanisms contributing to the incidence of fever-associated seizures in children. The delayed effect of cold exposure, peaking at 72–96 h, is consistent with the typical incubation period of respiratory viral infections. This pattern supports the hypothesis that cold stress primarily affects the incidence of pediatric seizures by increasing susceptibility to viral infections, which subsequently trigger fever and seizures. Potential pathways include compromised mucosal immunity due to cold exposure, which facilitates viral entry, and behavioral factors such as increased indoor crowding during colder weather, which enhances viral transmission (Eccles 2002; Burbank 2023).

In contrast, the mechanisms for heat exposure appear more complex than those for cold exposure, with effects varying by both time and intensity. UTCI levels around 24.2 °C (75th percentile), which are classified as No Thermal Stress by the standard UTCI scale, showed significant cumulative effects. This primary risk peak at 24.2 °C likely reflects the narrower pediatric-specific neutral range (6.4–21.5 °C) identified by Huang et al., (Huang et al. 2021) where temperatures perceived as comfortable for adults can still impose a physiological thermal load on children.

Furthermore, the hourly temporal resolution of our study likely contributed to detecting these associations compared to previous studies using daily averages. In Japan, largely characterized by a humid subtropical climate (Cfa), high humidity can sharply elevate the UTCI even at moderate ambient temperatures. These humidity-driven heat stresses, which might be masked in daily mean air temperature models, appear to particularly affect the pediatric population. This potentially explains why significant risks were observed even at UTCI 24.2 °C—a level considered neutral in other climate zones for adults, but one that may impose a critical physiological load on children in a humid environment. These findings may reflect a combination of direct thermoregulatory stress and delayed effects beyond 72 h possibly associated with summer-specific viral infections. The apparent decline in risk at extreme temperatures (above 33.1 °C) is likely explained by protective behavioral adaptations, such as increased time indoors in air-conditioned environments, which reduce direct exposure. However, this interpretation requires caution because of the wider CIs associated with limited statistical power at these extremes.

This study introduced several methodological advances. First, our use of the UTCI provides a more comprehensive assessment of thermal comfort than traditional temperature metrics by integrating multiple meteorological factors that affect human heat balance (Jendritzky et al. 2012; Błażejczyk et al. 2013). To the best of our knowledge, this study is the first to apply UTCI in research on fever-associated seizures in children and analyze the association between thermal stress and seizures at an hourly resolution. Second, the hourly temporal resolution suggested acute exposure–response relationships that were missed by previous daily analyses, particularly the immediate effects of heat exposure, although it did not reach statistical significance. Third, our 144-h lag period comprehensively evaluated immediate physiological and delayed infection-mediated pathways. The time-stratified case-crossover design with the DLNM effectively controlled for individual-level confounders and temporal trends, while flexibly modeling non-linear and delayed associations (Gasparrini et al. 2010). The use of ambulance dispatch data provided population-based coverage with accurate temporal information and objective temperature measurements by EMS personnel.

However, several limitations warrant consideration. First, our case definition based on EMS data may have introduced misclassification bias because body temperature measured at EMS contact may not reflect the body temperature at seizure onset owing to antipyretic use or post-ictal changes. Second, we lacked individual-level data on potential effect modifiers, such as underlying medical conditions, medication use, and socioeconomic factors. While the case-crossover design controls for time-invariant individual factors, we could not examine effect modification by these characteristics. Third, the reliance on pre-hospital data meant that we could not characterize the heterogeneity of cases or differentiate seizures by their underlying etiology (e.g., those secondary to central nervous system infections or heat stroke versus from more benign FSs), by clinical type (e.g., simple vs. complex), or by specific etiology (e.g., viral agents, genetic predispositions). This limitation precluded any subgroup analysis to examine whether the association between UTCI exposure and fever-associated seizures varies across these different clinical presentations. Fourth, the wide CIs, particularly at extreme temperatures, reflect limited statistical power for extreme exposures. The seasonal stratification analysis was underpowered, which prevented formal statistical testing of seasonal effect modification. Additionally, we could not directly measure viral infections, relying instead on temporal patterns to infer infection-mediated pathways. Therefore, the viral-mediated mechanism remains a hypothesis generated from the observed lag structures and warrants direct validation in future studies incorporating clinical virological data.

Our findings have important implications for both environmental public health and clinical management of fever-associated seizures in children. The identification of specific UTCI thresholds and lag structures provides an evidence base for targeted, climate-informed interventions. The results demonstrate that in vulnerable East Asian pediatric populations, risk is bimodally distributed —peaking at both Moderate Cold Stress and Moderate Heat Stress (including upper range of No Thermal Stress) — which challenges the conventional focus on Strong Heat alone.

Given the thermal stress effects on FAS, public health strategies should focus on monitoring delayed effects rather than immediate clinical interventions. Preventive measures should address potential infection-mediated risks during the 3–4-day window following Moderate Cold and Moderate Heat (including the upper range of No Thermal Stress) events. Our findings indicate that even within the UTCI range typically considered comfortable for adults (around 24.2 °C), there is a significant cumulative risk for pediatric seizures. Sustained monitoring for delayed fever and subsequent seizures remains important even under seemingly mild conditions, as these levels may still impose a physiological load on children or coincide with increased viral transmission.

Future research should include multi-city and multi-climate-zone studies to assess generalizability and identify population-specific susceptibility factors. Developing real-time early warning systems or forecasting models that combine UTCI predictions with epidemiological and virological data could significantly enhance preparedness. Further investigation of the long-term neurodevelopmental implications of fever-associated seizures in children, particularly in relation to environmental exposures and climate change, is also warranted.

## Conclusions

This study demonstrated a bimodal association between hourly thermal stress and fever-associated seizures in children.

Moderate Cold Stress (−1.6 °C) was associated with a significant cumulative risk peaking between 72–96 h, while Moderate Heat Stress including upper range of No Thermal Stress (24.2 °C and 28.7 °C) showed a delayed cumulative risk peaking at approximately 100 h. This delayed pattern, contrasted with the more immediate but statistically non-significant risk associated with Strong Heat, suggests that most pediatric fever-associated seizures are likely driven by infection-mediated pathways rather than direct thermoregulatory failure.

The use of high-resolution bioclimatic indices like the hourly UTCI was essential for capturing acute thermal vulnerabilities that are often masked by daily averages. These findings provide a basis for developing temperature-specific early warning systems that monitor for delayed risks.

While providing evidence for a Japanese population, this study is limited by its single-center design (Okayama, Japan) and the use of ambient meteorological data as a proxy for personal exposure. Future multi-city studies integrating clinical virological data are required to validate the hypothesized biological mechanisms across diverse climatic zones.

## Data Availability

The meteorological data used in this study (ERA5-Land reanalysis) are publicly available through the Copernicus Climate Data Store (https://cds.climate.copernicus.eu/). The individual-level ambulance transport data used in this study were provided by the Okayama City Fire Department under a data use agreement and cannot be shared publicly due to legal and privacy restrictions. Statistical code used for analyses (Stata and R) are available from the corresponding author upon reasonable request.
